# Transcriptional Activation of Low-Density Lipoprotein Receptor Gene by DJ-1 and Effect of DJ-1 on Cholesterol Homeostasis

**DOI:** 10.1371/journal.pone.0038144

**Published:** 2012-05-30

**Authors:** Shiori Yamaguchi, Takuya Yamane, Kazuko Takahashi-Niki, Izumi Kato, Takeshi Niki, Matthew S. Goldberg, Jie Shen, Kenji Ishimoto, Takefumi Doi, Sanae M. M. Iguchi-Ariga, Hiroyoshi Ariga

**Affiliations:** 1 Graduate School of Pharmaceutical Sciences, Hokkaido University, Sapporo, Japan; 2 Graduate School of Agriculture, Hokkaido University, Sapporo, Japan; 3 Center for Neurologic Diseases, Brigham & Women’s Hospital, Program in Neuroscience, Harvard Medical School, Boston, United States of America; 4 Graduate School of Pharmaceutical Sciences, Osaka University, Suita, Japan; University of Hong Kong, Hong Kong

## Abstract

*DJ-1* is a novel oncogene and also causative gene for familial Parkinson’s disease *park7*. DJ-1 has multiple functions that include transcriptional regulation, anti-oxidative reaction and chaperone and mitochondrial regulation. For transcriptional regulation, DJ-1 acts as a coactivator that binds to various transcription factors, resulting in stimulation or repression of the expression of their target genes. In this study, we found the low-density lipoprotein receptor (LDLR) gene is a transcriptional target gene for DJ-1. Reduced expression of LDLR mRNA and protein was observed in DJ-1-knockdown cells and DJ-1-knockout mice and this occurred at the transcription level. Reporter gene assays using various deletion and point mutations of the LDLR promoter showed that DJ-1 stimulated promoter activity by binding to the sterol regulatory element (SRE) with sterol regulatory element binding protein (SREBP) and that stimulating activity of DJ-1 toward LDLR promoter activity was enhanced by oxidation of DJ-1. Chromatin immunoprecipitation, gel-mobility shift and co-immunoprecipitation assays showed that DJ-1 made a complex with SREBP on the SRE. Furthermore, it was found that serum LDL cholesterol level was increased in DJ-1-knockout male, but not female, mice and that the increased serum LDL cholesterol level in DJ-1-knockout male mice was cancelled by administration with estrogen, suggesting that estrogen compensates the increased level of serum LDL cholesterol in DJ-1-knockout female mice. This is the first report that DJ-1 participates in metabolism of fatty acid synthesis through transcriptional regulation of the *LDLR* gene.

## Introduction

The *DJ-1* gene has been identified by us as a novel oncogene that transforms NIH3T3 cells in cooperation with the activated *ras* gene [1] and was later found to be a causative gene for familial Parkinson’s disease *park7* [2]. DJ-1 is expressed ubiquitously in cultured cells and tissues and is localized in the cytoplasm, nucleus and mitochondria [1], [3]–[6]. DJ-1 has multiple functions, including transcriptional regulation [7]–[15], anti-oxidative stress function [3], [16]–[20], chaperone [4], [21], protease [22]–[24] and mitochondrial regulation [25]–[28]. DJ-1 binds to various signaling proteins such as PTEN [29], [30], ASK1 [31], [32], HIPK1 [33] and Daxx [34] to affect their signaling cascades, leading to progression of cell growth and inhibition of cell death. For its role in transcriptional regulation, DJ-1 binds to various transcription factors, including inhibitors for androgen receptor [7], [8], p53 [9], [14], polypyrimidine tract-binding protein-associated splicing factor (PSF) [10] and Keap1, an inhibitor for nuclear factor erythroid-2 related factor 2 (Nrf2) [11], to modulate their transcriptional activity, resulting in various effects on cell functions. It is therefore thought that loss of and excess activation of DJ-1 render the onset of neurodegenerative diseases such as Parkinson’s disease and cancer, respectively.

We previously searched for genes whose expression was changed in DJ-1-knockdown cells compared to that in parental cells by using a DNA microarray, and we identified many candidate genes, including the low-density lipoprotein receptor (LDLR) gene [35]. LDLR is cell surface protein involved in receptor-mediated endocytosis of a specific ligand, low-density lipoprotein (LDL). LDL is then transferred into the lysosome, where it is degraded and cholesterol is produced by microsomal enzyme 3-hydroxy-3-methylglutaryl coenzyme A (HMG CoA) reductase. The level of LDLR is related to pathogenesis of lipidosis and type 2 diabetes mellitus, and mutations in the *LDLR* gene cause the autosomal dominant disorder familial hypercholesterolemia. Expression of the *LDLR* gene is activated by sterol regulatory element binding protein (SREBP), which binds to the sterol regulatory element (SRE) on the *LDLR* gene promoter in cooperation with Sp1 [36]–[39]. The SRE is also present in genes for HMG CoA reductase and HMG CoA synthetase and acts as a positive element that responds to reduction of the cholesterol level in cells. It is also known that estrogen stimulates the promoter activity of the LDLR promoter [40].

In this study, we found using a cell culture system and DJ-1-knockout mice that DJ-1 stimulates expression of the *LDLR* gene at the transcriptional level by association with SREBP and affects the level of serum LDL cholesterol in male mice.

## Results

### Reduced Expression of Low-density Lipoprotein Receptor Gene in Dj-1-Knodown Cells and Knockout Mice

**Figure 1:**
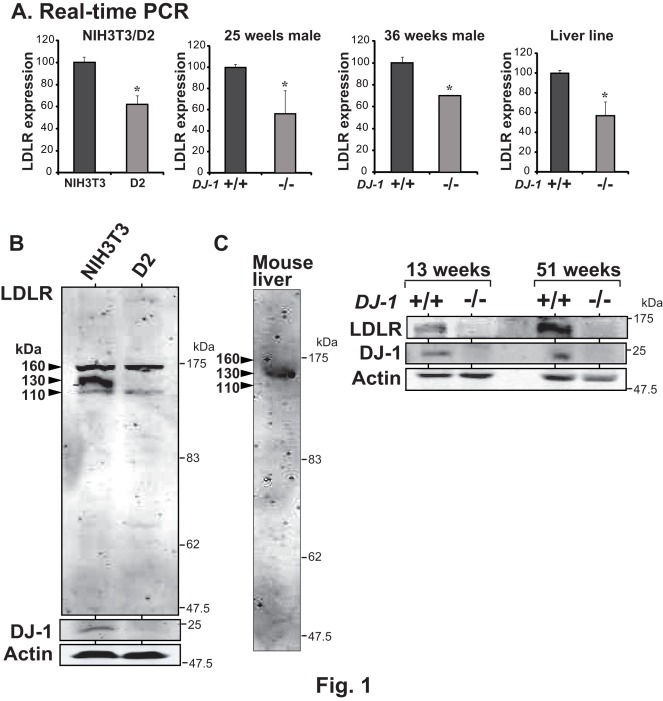
Reduction of *LDLR* gene expression in DJ-1-knockdown cells and DJ-1-knockout mice. A. Relative mRNA levels of LDLR were examined by quantitative RT-PCR (real-time PCR) in NIH3T3 and its DJ-1-knockdown D2 cells, in livers from wild-type and DJ-1-knockout mice at 25 and 36 weeks of age and in the established liver cell line from wild-type and DJ-1-knockout mice. Actin or GAPDA mRNA was also amplified by real-time PCR as loading controls. Statistical analyses were carried out using Student’s *t*-test. Number of experiments (n) is 3. B and C. Proteins extracted from NIH3T3 and D2 cells (B) and from livers of wild-type and DJ-1-knockout mice at 13 and 51 weeks of age (right panels in C) were analyzed by Western blotting with anti-LDLR, anti-DJ-1 and β-actin antibodies. β-actin was used as a loading control. Proteins from the liver of wild-type mouse at 25 weeks of age were analyzed by Western blotting with an anti-LDLR antibody (left panel in C).

We have screened genes whose expression was reduced in D2 cells, which are DJ-1-knocked down NIH3T3 cells, compared to that in parental NIH3T3 cells by using a DNA microarray, and the low-density lipoprotein receptor (LDLR) gene was found to be a candidate gene [35]. To confirm this, total RNA was extracted from D2 and NIH3T3 cells and the expression levels of LDLR, DJ-1 and actin mRNA were examined by semi-quantitative RT-PCR (data not shown) and by quantitative real-time PCR. Actin mRNA was used as a loading control. As shown in Figure 1A, the expression levels of LDLR and DJ-1 mRNAs in D2 cells were reduced to about 60% of those in NIH3T3 cells. To examine whether reduced expression of LDLR mRNA occurs in mice, RNA was extracted from the liver of DJ-1-knockout and normal mice at 25 weeks and 36 weeks of age and quantitative real-time PCR was carried out. As in the case of D2 cells, about 50% and 30% reduction of LDLR mRNA expression was found in DJ-1-knockout mice at 25 weeks and 36 weeks of age, respectively. Furthermore, liver cell lines from DJ-1-knockout and normal mice were established after liver cells from newborn male mice had been immortalized by SV40 T antigen, and the expression level of their mRNA was examined by quantitative real-time PCR. Again, reduced expression of LDLR mRNA was found in DJ-1-knockout cells. Expression levels of LDLR and DJ-1 in NIH3T3 and D2 cells and in the liver from DJ-1-knockout mice were then examined by Western blotting. Three bands corresponding to LDLR were observed in NIH3T3 and these bands are known to be differentially glycosylated LDLR. Although intensity of all of the three bands was reduced in D2 cells, a band with 130 kDa was almost disappeared (Figure 1B). Only a band of LDLR with 130 kDa was, on the other hand, observed in the liver of mice at various ages, and the result of mice at 25 weeks of age was shown (Figure 1C, left panel). As in the case of mRNA levels, reduced levels of LDLR were found in DJ-1-knockout mice at 13 weeks and 51 weeks of age (Figure 1C, right panel).

**Figure 2:**
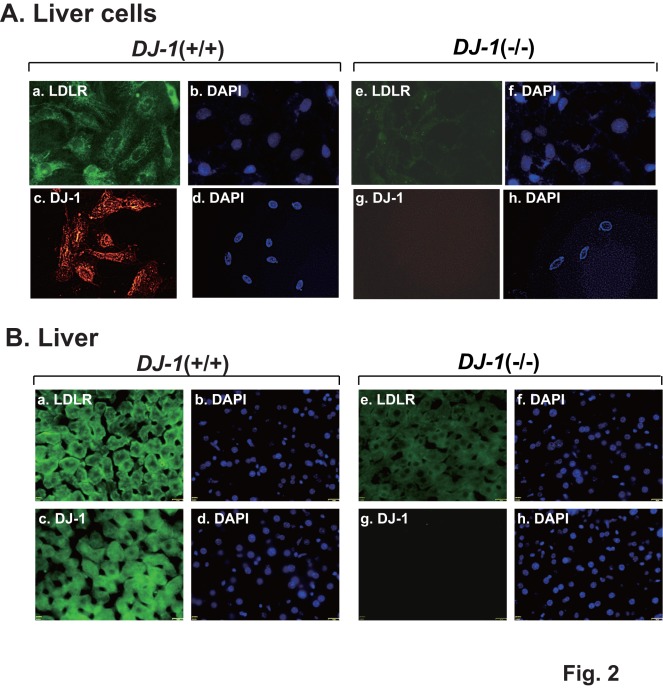
Reduction of LDLR expression in DJ-1-knockout cells and in DJ-1-knockout mice. A. Mouse liver cells from wild-type and DJ-1-knockout mice were immunostained with anti-LDLR and anti-DJ-1 antibodies. The cells were then reacted with an FITC-conjugated anti-rabbit IgG or with a rhodamine-conjugated anti-rabbit IgG for 1 hr, and their nuclei were stained with DAPI. The cells were then observed under a fluorescent microscope as described in Materials and methods. B. Liver sections from wild-type and DJ-1-knockout mice were immunostained with anti-LDLR and anti-DJ-1 antibodies and visualized after reaction with an FITC-conjugated anti-rabbit IgG as described in Materials and methods. Nuclei were also stained with DAPI.

The expression levels of LDLR and DJ-1 were further examined by an immunostaining method. Liver cell lines and liver sections from DJ-1 (+/+) and DJ-1 (−/−) mice were stained with anti-LDLR and anti-DJ-1 antibodies. Nuclei were also stained with DAPI. As shown in Figure 2, the expression levels of LDLR in liver cells and in liver from DJ-1 (−/−) mice were significantly reduced. These results indicate that reduced or no expression of DJ-1 rendered reduced expression of the *LDLR* gene.

### Stimulation of LDLR Promoter Activity by DJ-1

**Figure 3:**
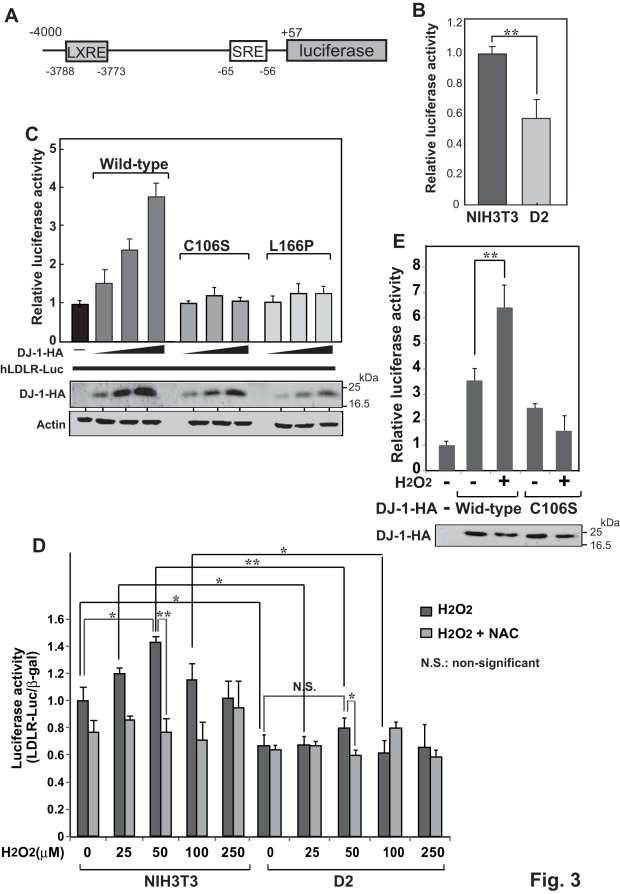
Stimulation of promoter activity of the *LDLR* gene by DJ-1. A. Schematic drawing of the reporter gene comprised of the LDLR promoter and the luciferase gene. B. NIH3T3 and D2 cells in 24-well dishes were transfected with 0.75 µg of pGL4.10-hLDLR and 0.25 µg of pCMV-β-gal. Forty-eight hrs after transfection, cell extracts were prepared and their luciferase activity was measured as described in Materials and methods. Statistical analyses were carried out using Student’s *t*-test. Number of experiments (n) is 3. C. D2 cells in a 6-well dish were transfected with 0.75 µg of pGL4.10-hLDLR and 0.25 µg of pCMV-β-gal together with 0.25, 0.75 and 1.0 µg of pEF-DJ-1-HA (wild-type, C106 and L166 mutants of DJ-1). Forty-eight hrs after transfection, cell extracts were prepared and their luciferase activity was measured as described in Materials and methods. The expression level of DJ-1-HA and actin in cell extracts was analyzed by Western blotting with an anti-HA antibody. Number of experiments (n) is 4. D. NIH3T3 and D2 cells in a 6-well plat were transfected with 0.75 µg of pGL4.10-hLDLR and 0.25 µg of pCMV-β-gal. At 48 hrs after transfection, cells were exposed to various concentration of H_2_O_2_ together to 2 mM N-acetylcysteine for 1 hr and their luciferase activity was measured. Number of experiments (n) is 4. E. D2 cells in a 6-well dish were transfected with 0.75 µg of pGL4.10-hLDLR and 0.25 µg of pCMV-β-gal together with 1.0 µg of pEF-DJ-1-HA (wild-type and C106). Forty-eight hrs after transfection, cells were exposed to 100 µM H_2_O_2_ for 1 hr and their luciferase activity was measured. The expression level of DJ-1 in cell extracts was analyzed by Western blotting with an anti-HA antibody. Number of experiments (n) is 4. Statistical analyses in Figure 6C, 6D and 6E were carried out using the Tukey-Kramer test.

To examine the effect of DJ-1 on LDLR gene promoter activity, the upstream region of the *LDLR* gene spanning −4000 to +57 linked to the *luciferase* gene (pGL4.10-hLDLR 200) [41] was transfected into D2 and NIH3T3 cells and its luciferase activity was measured. The upstream region used contains two important elements, LXRE and SRE (Figure 3A). As shown in Figure 3B, luciferase activity in D2 cells was reduced to 58% compared to that in NIH3T3 cells, suggesting that promoter activity of the *LDLR* gene was attenuated in DJ-1-knockdown cells. To further assess the effect of DJ-1 on LDLR promoter activity, D2 cells were transfected with pGL4.10-hLDLR 200 together with various amounts of expression vectors for wild-type DJ-1, C106S and L166P mutants of DJ-1, and the luciferase activity was measured (Figure 3C). C106S and L166P mutants of DJ-1 are substitution mutants from cysteine at amino acid number 106 (C106) to serine and from leucine at amino acid number 166 to proline, respectively. Since C106 of DJ-1 is the most sensitive amino acid residue toward oxidative stress and an essential amino acid for DJ-1′s function, C106S DJ-1 has no or little activity [2], [4], [16]. L166P DJ-1 has been found in patients with familial Parkinson’s disease [2]. The results showed that while wild-type DJ-1 stimulated luciferase activity in a dose-dependent manner, neither C106S nor L166P mutants of DJ-1 stimulated luciferase activity, suggesting that stimulation of LDLR promoter activity needs a wild-type conformation of DJ-1 and that oxidative stress affects stimulating activity toward the LDLR promoter.

To address the effect of oxidative stress on DJ-1-stimulated LDLR promoter activity, NIH3T3 and D2 cells were transfected with pGL4.10-hLDLR 200. At 48 hrs after transfection, cells were exposed to various concentrations of H_2_O_2_ for 1 hr together with or without N-acetylcysteine (NAC), an antioxidant, and their luciferase activity was measured (Figure 3D). Luciferase activity in NIH3T3 cells was increased up to 50 µM H_2_O_2_ in a dose-dependent manner and then decreased at 100 and 250 µM H_2_O_2_ exposure. These effects of H_2_O_2_ on luciferase activity were not observed in NIH3T3 cells that had been treated with NAC. In D2 cells exposed to 0–250 µM H_2_O_2_, on the other hand, no stimulation of luciferase activity was observed regardless of the presence or absence of NAC. Stimulation curve of luciferase activity in H_2_O_2_-treated NIH3T3 cells is similar to that observed in DJ-1-activated tyrosine hydroxylase promoter activity in H_2_O_2_-treated SH-SY5Y cells as described previously [13]. In this case, oxidative status of C106 of DJ-1 determined the stimulation level of tyrosine hydroxylase promoter activity by DJ-1 [13]. Furthermore, D2 cells were transfected with pGL4.10-hLDLR 200 together with expression vectors for wild-type DJ-1 and C106S mutant of DJ-1. At 48 hrs after transfection, cells were exposed to 100 µM H_2_O_2_ and the luciferase activity was measured (Figure 3E). The results showed that luciferase activity was increased by wild-type DJ-1 and further increased by H_2_O2 exposure. The effect of C106S DJ-1 on luciferase activity was weaker than that of wild-type DJ-1, and no stimulation by C106S DJ-1 was observed after cells were exposed to H_2_O_2_. These results suggest that stimulation of LDLR promoter activity by H_2_O_2_ exposure depends on oxidative status of C106 of DJ-1, but not on simple oxidation to cells.

**Figure 4:**
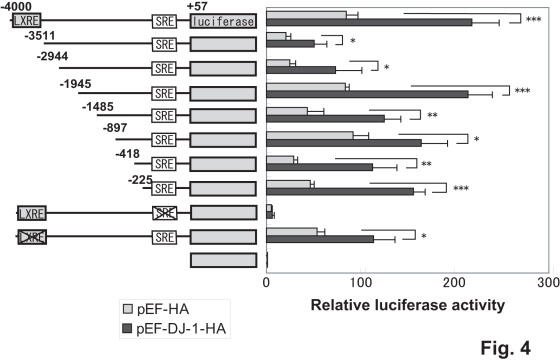
Identification of the target region for DJ-1 in the LDLR promoter. Various deletion constructs of the LDLR promoter linked to the *luciferase* gene were constructed and transfected into D2 cells together with pEF or pEF-DJ-1-HA as described in the legend of Figure 2. Forty-eight hrs after transfection, luciferase activity was measured. Statistical analyses were carried out using Student’s *t*-test. Number of experiments (n) is 3.

To determine the region targeted by DJ-1, various deletion constructs of the LDLR promoter linked to the *luciferase* gene were constructed and they were transfected into D2 cells with or without an expression vector for DJ-1. As shown in Figure 4, various deletions up to −225 from a transcriptional start site similarly reacted to DJ-1, suggesting that the region −225 to +57 contains the DJ-1-responsive region. Since *LDLR* gene expression has been reported to be regulated by two elements, the sterol regulatory element (SRE) and liver X receptor response element (LXRE), and the region −225 to +57 contains the SRE [36], [37], [41], the reporter construct containing either mutation of SRE or LXRE was transfected into D2 cells. The results showed that while LXRE mutation did not affect the response to DJ-1, SRE mutation abolished the response to DJ-1 (Figure 4), suggesting that the SRE is a target site for DJ-1.

### Association of DJ-1 with the Sterol Regulatory Element

**Figure 5:**
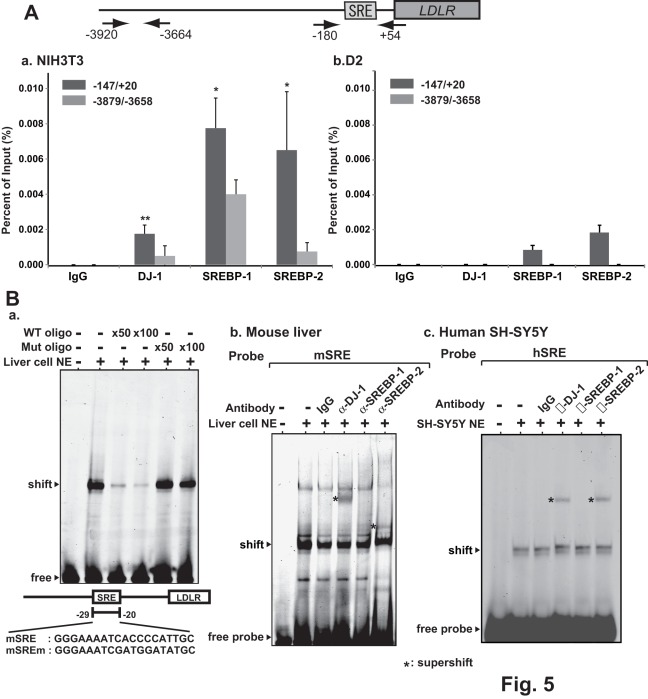
Association of DJ-1 and SREBP with the LDLR promoter. A. Chromatin immunoprecipitation assays were carried out using chromatin prepared from NIH3T3 (a) and D2 (b) cells. Chromatin was immunoprecipitated with anti-DJ-1, anti-SREBP-1 and anti-SREBP-2 antibodies or non-specific IgG. After extraction of DNA from precipitated chromatin, two regions spanning −180 to +54 and spanning −3920 to +54 were amplified by real-time PCR with specific primers and with amplified DNA as described in Materials and methods. Statistical analyses were carried out using Student’s *t*-test. Number of experiments (n) is 3. B. Gel-mobility shift assays were carried out using nuclear extracts from mouse liver and SH-SY5Y cells with IRDye800-labeled SRE oligonucleotide as a probe. a. NIH3T3 nuclear extracts were mixed with 50 and 100-times molar ratio of wild-type and mutated oligonucleotide compared to that of IRDye800-labeled SRE and subjected to gel-mobility shift assays. b and c. Mouse liver cell (b) or D2 cell (c) nuclear extracts were first reacted with the IRDye800-labeled SRE probe for 30 min at 0°C and then with an anti-DJ-1 antibody, anti-SREBP-1 antibody, anti-SREBP-2 antibody or IgG, and then separated on 4% polyacrylamide gel as described in Materials and methods.

SREBP-1 and SREBP-2 are proteins that bind to the SRE. To examine the association of DJ-1 with the SRE, chromatin immunoprecipitation assays were carried out. Chromatin extracted from NIH3T3 cells was reacted with non-specific IgG or anti-DJ-1, anti-SREBP-1 and anti-SREBP-2 antibodies, and two regions spanning −3,920 to −3,664 and spanning −180 to +54 were amplified by real-time PCR with specific primers and with precipitated DNA as a template. As shown in Figure 5A-a, anti-DJ-1, anti-SREBP-1 and anti-SREBP-2 antibodies but not IgG specifically precipitated the region spanning −180 to +54 and small amounts of amplification in the region spanning −3,920 to −3,664 were observed, indicating that DJ-1, SREBP-1 and SREBP-2 bound to this region. Chromatin immunoprecipitation assays were also carried out using chromatin extracted from DJ-1-knockdown D2 cells (Figure 5A-b). The results showed that anti-SREBP-1 and anti-SREBP-2 antibodies, but not the anti-DJ-1 antibody, precipitated the region spanning −180 to +54 and that the levels of precipitated DNA from D2 chromatin were lower than those from NIH3T3 chromatin. Gel photos showing the final PCR products are also shown in Figure S1.

To further assess the binding of DJ-1 with the SRE, gel-mobility shift assays were carried out using nuclear extracts from mouse liver cells and Cy5.5-labeled SRE as a probe. DNA-protein complex was found on the SRE, and a shifted band on the SRE disappeared after addition of excess amounts of non-labeled SRE oligonucleotide but not mutated oligonucleotide (Figure 5B-a), indicating that DNA-protein complex was specific to the SRE. After addition of non-specific IgG or anti-DJ-1, anti-SREBP-1 and anti-SREBP-2 antibodies to reaction mixtures, the specific band was supershifted with anti-DJ-1 and anti-SREBP-2 antibodies but not with IgG and with an anti-SREBP-1 antibody (Figure 5B-b). Supershift assays were also carried out using human SH-SY5Y nuclear extracts, and anti-DJ-1 and anti-SREBP-2 antibodies but not the anti-SREBP-1 antibody supershifted a band of the SRE-protein complex (Figure 5B-c). Different mobility of the band supershifted by the anti-SREBP-2 antibody in liver and SH-SY5Y cells may be different origins of two cell limes. These results indicate that protein complexes containing DJ-1, SREBP-1 and SREBP-2 bind to the SRE. To examine direct interaction of DJ-1 with SREBP1 or SREBP2, pull-down experiments were carried out. GST-DJ-1 purified from *E. coli* was reacted with ^35^S-labeled SREBP-1 or SREBP-2, which had been synthesized using a reticulocyte lysate *in vitro*. The results showed that neither SREBP-2 nor SREBP-1 directly bound to DJ-1 (Figure S2). Gel-mobility shift assays were then carried out using recombinant human DJ-1 and Cy5.5-labeled wild-type SRE and LXRE as probes, and the result using an SRE probe was shown (Figure S3). No binding of DJ-1 to the SRE was observed.

**Figure 6:**
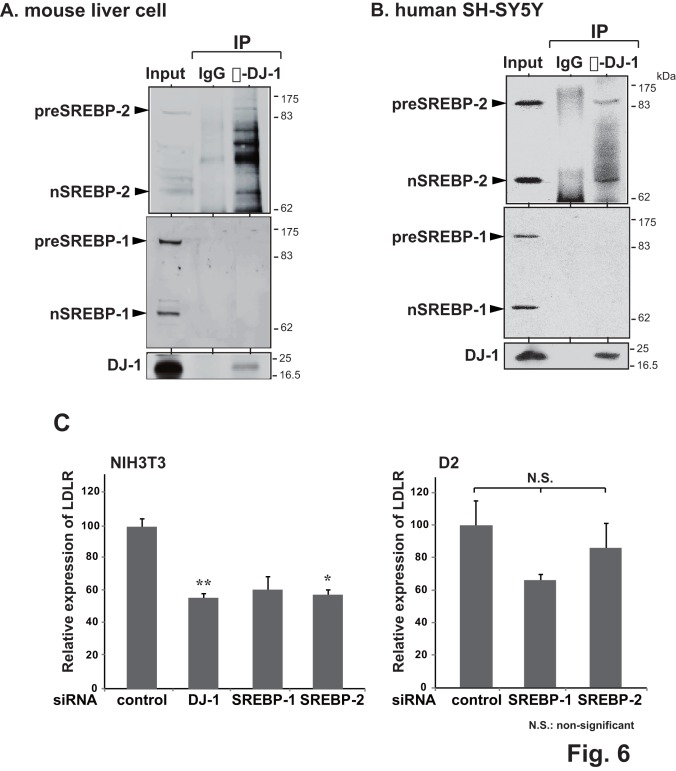
Association of DJ-1 with SREBP-2 and involvement of DJ-1 in LDLR expression. A and B. Proteins in mouse liver cell (A) or SH-SY5Y cell (B) nuclear extracts were immunoprecipitated with an anti-DJ-1 antibody or IgG. Immunoprecipitates were then analyzed by Western blotting with anti-SREBP-1, anti-SREBP-2 and anti-DJ-1 antibodies. PreSREBP-2 and preSREPB-1 indicate precursor SREBP-2 and precursor SREPB-1, and nSREBP-2 and nSREPB-1 indicate cleaved SREBP-2 and cleaved SREPB-1, respectively. C. NIH3T3 and D2 cells were transfected with siRNAs targeting DJ-1, SREBP-1 and SREBP-2 and with non-specific siRNA. At 48 hrs after transfection, expression levels of LDLR and actin mRNA were examined by real-time PCR and relative expression of LDLR against actin was shown. Statistical analyses were carried out using the Tukey-Kramer test.

Since DJ-1 does not directly bind to DNA (Figure S3) and since it has been reported that SREBP-1/SREBP-2 directly binds to the SRE and that SREBP-2 and SREBP-1 make heterodimer [42], it is possible that DJ-1 binds to the SRE in association with SREBP-1/SREBP-2 via unknown protein(s). To examine this possibility, protein extracts from mouse liver cells and SH-SY5Y cells were immunoprecipitated with an anti-DJ-1 antibody or non-specific IgG and precipitates were analyzed by Western blotting with anti-SREBP-1, anti-SREBP-2 and anti-DJ-1 antibodies. SREBP-1 and SREBP-2 are known to be cleaved from precursor forms to be activated. As shown in Figure 6A and 6B, the anti-DJ-1 antibody precipitated both precursor and cleaved forms of SREBP-2 but not those of SREBP-1, indicating association of DJ-1 with SREBP-2.

To confirm roles of DJ-1, SREBP1 and SREBP2 in transcriptional activation of the LDLR gene, NIH3T3 cells were transfected with siRNAs targeting *DJ-1*, *SREBP-1* and *SREBP-2* and with non-specific siRNA, and the expression level of LDLR mRNA was examined by real-time PCR at 48 hrs after transfection. As shown in Figure 6C, siRNAs for *DJ-1* and *SREBP-2*, but not for *SREBP-1*, significantly reduced expression levels of LDLR mRNA. Furthermore, when DJ-1-knockdown D2 cells were transfected with siRNAs targeting *SREBP-1* and *SREBP-2* and with non-specific siRNA, the expression levels of LDLR mRNA were not significantly affected. These results suggest that the DJ-1/SREBP-2 complex binds to the SRE on the LDLR promoter to activate its promoter activity and that SREBP-1 also binds to the SRE without complex formation with DJ-1. Since the binding level of SREBP-1 to the LDLR promoter is reduced in DJ-1-knockdown cells, DJ-1 may affect binding activity of SREBP-1 by unknown mechanism.

### Serum Cholesterol Levels in DJ-1-knockout Mice

**Figure 7:**
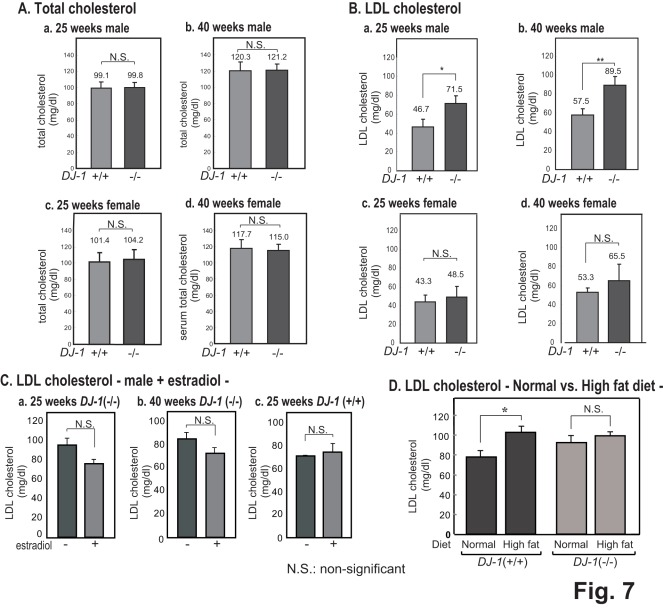
Total cholesterol and LDL levels in wild-type and DJ-1-knockout mice. A. Total cholesterol levels in wild-type and DJ-1-knockout mice at 25 and 40 weeks of age were measured by using a cholesterol E kit (Wako Pure Chemical). Number of experiments (n) is 5. B. LDL cholesterol levels in wild-type and DJ-1-knockout mice at 25 and 40 weeks of age were measured by using an LDL-C.M. kit (Wako Pure Chemical). Number of experiments (n) is 5–7. C. DJ-1-knockout male mice at 25 and 40 weeks of age and wild-type male mice at 25 weeks of age were administered 5 µg/g body weight of estradiol for every 6 days, and their LDL cholesterol levels were measured. Number of experiments (n) is 4–7. D. Wild-type and DJ-1-knockout mice at 13 weeks of age were fed with a high-cholesterol diet, and their serum LDL cholesterol levels were measured as described above. Number of experiments (n) is 4–7. Statistical analyses of Figs. 7A-7D were carried out using Student’s *t*-test.

Since the expression level of LDLR is related to pathogenesis of lipidosis and type 2 diabetes mellitus and since DJ-1 regulates the LDLR expression as described above, the effect of DJ-1 on cholesterol levels was examined using DJ-1-knockout mice. First, the total cholesterol amounts in serum from wild-type and DJ-1-kickout mice were measured. The total cholesterol levels of male and female mice at 25 and 40 weeks of age tended to increase with age, but there were no significant difference between wild-type and DJ-1-knockout mice regardless of age or sex (Figure 7A). Next, the serum LDL cholesterol level was examined. Although the serum LDL cholesterol level in female mice at 25 and 40 weeks of age was not significantly changed between wild-type and DJ-1-knockout mice, it was found that the level in male mice was significantly increased in DJ-1-knockout mice at both ages compared to that in wild-type mice (Figure 7B), suggesting that DJ-1 affects metabolism of LDL cholesterol in male mice. To explain different effects of DJ-1 inactivation on serum LDL cholesterol levels in male and female mice, estrogen was administered to DJ-1-knockout male mice at 25 and 40 weeks of age and serum LDL cholesterol levels were measured. Wild-type male mice at 25 weeks of age were also administered estrogen as a negative control. As shown in Figure 7C, there were no significant differences of serum LDL cholesterol levels in DJ-1-knockout male mice at both ages with or without administration of estrogen. These results suggest that estrogen is one of factors that influence the effect of DJ-1 on the serum LDL cholesterol level. It has been reported that when LDLR-knockout mice were fed with a high-cholesterol diet, they had a three-fold higher concentration of the serum LDL cholesterol than that in mice fed with an ordinary diet, resulting in atherosclerosis [43]. To examine the effect of diets on the serum LDL cholesterol level of DJ-1-kockout mice, wild-type and DJ-1-kockout male mice at 13 weeks of age were fed with a high-cholesterol diet, and their serum LDL cholesterol levels were measured. While the serum LDL cholesterol level of wild-type mice was increased after mice were fed with the high-cholesterol diet, no significant change was observed in DJ-1-knockout mice. These results suggest the specific effects of DJ-1 in cholesterol homeostasis.

## Discussion

In this study, we first found that DJ-1 positively regulates *LDLR* gene expression at the transcriptional level through association of SREBP on the SRE located in the LDLR promoter. Reduced expression of the *LDLR* gene was observed in DJ-1-knockdown cells, DJ-1-knockout cells and DJ-1-knockout mice. We then found that the serum LDL cholesterol level is increased in DJ-1-knockout male mice compared to that in wild-type mice. This is the first finding of participation of DJ-1 in cholesterol metabolism.

Deletion and point mutation analysis of the LDLR promoter showed that of two known elements, LXRE and SRE, which are important for LDLR expression [36], [37], [41], the SRE was found to be a target for DJ-1. SREBP-1 and SREBP-2 recognize the same sequence of the SRE (see a recent review 43, original references therein). While SREBP-2 is ubiquitously expressed in tissues, SREBP-1 is preferentially expressed in the liver and adrenal gland [44]. Although chromatin immunoprecipitation (ChIP) assays using NIH3T3 and its DJ-1 knockdown D2 chromatin showed that anti-DJ-1, anti-SREBP-1 and anti-SREBP-2 antibodies precipitated the region containing the SRE (Figure 5A), gel-mobility shift and co-immunoprecipitation assays using mouse liver and SH-SY5Y cell extracts showed that anti-DJ-1 and anti-SREBP-2 antibodies, but not an anti-SREBP-1 antibody, supershifted a band corresponding to the SRE-protein complex and that DJ-1 is associated with SREBP-2 but not with SREBP-1 (Figure 5B and 6, respectively). Direct interaction of DJ-1 with SREBP2 and SREBP-1 was not observed in pull-down assays (Figure S2). DJ-1 does not directly bind to DNA (Fig. S3), and SREBP-1/SREBP-2 directly binds to the SRE [42]. siRNAs targeting *SREBP-2* and *SREBP-1* do not significantly reduce the expression level of LDLR mRNA in DJ-1-knockdown D2 cells (Figure 6C). These results suggest that the DJ-1/SREBP-2 complex binds to the SRE on the LDLR promoter to activate its promoter activity and that SREBP-1 also binds to the SRE without complex formation with DJ-1. Since the binding level of SREBP-1 to the LDLR promoter was reduced in DJ-1-knockdown cells (Figure 5A-b), DJ-1 may affect binding activity of SREBP-1 by unknown mechanism.

Stimulating activity of DJ-1 toward the LDLR promoter also depends on oxidative stress in cells expressing the normal level of DJ-1 (Figure 3D). LDLR promoter activity in NIH3T3 cells showed a biphasic pattern during course of H_2_O_2_ exposure: first increase and then decrease of activity, and this pattern is not observed in D2 cells. Furthermore, wild-type DJ-1 but not C106S DJ-1 activated LDLR promoter activity in an oxidative stress-dependent manner (Figure 3E). These results suggest that the oxidative status of C106 of DJ-1 affects LDLR promoter activity as in the case of DJ-1-activating tyrosine hydroxylase promoter activity [13].

Since SREBP-2 is a positive regulator for genes related to cholesterol metabolism, it would be interesting if the complex of DJ-1 with SREBP-2 also regulates transcription of other genes related to cholesterol metabolism. In microarray experiments, we have identified a gene encoding 24-dehydrocholesterol reductase (Dhcr24) whose expression was reduced in DJ-1-knockdown cells [35]. Since an SRE-like sequence is present in the promoter region of the *Dhcr24* gene, it is possible that the DJ-1/SREBP-2 complex positively regulates Dhcr24 expression, too.

**Figure 8:**
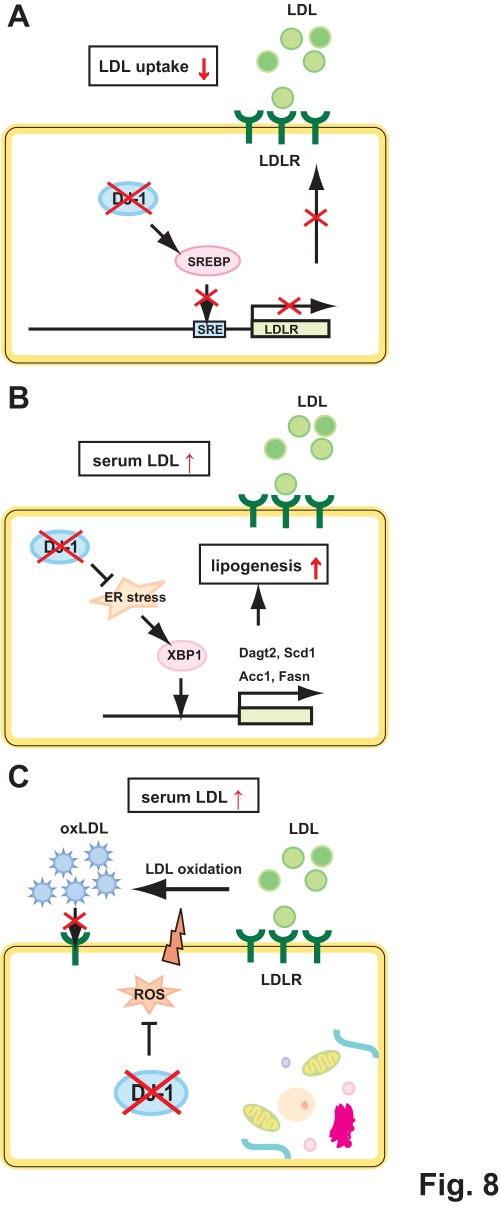
Model of increased serum LDL cholesterol in DJ-1-knockout mice.

In a latter part, we found that the total cholesterol level is not changed between wild-type and DJ-1-knockout mice regardless of gender or age. Since the total cholesterol level of wild-type mice is known to be in the range of 80–120 mg/dl [45]–[47] and that of DJ-1-knockout mice was within this range (Figure 7A), it is thought that DJ-1 expression does not affect the total cholesterol level. The serum LDL cholesterol level in DJ-1-knockout male mice was, however, significantly increased compared to that in wild-type male mice and there was no significant change in DJ-1-knockout female mice (Figure 7B), suggesting that DJ-1 participates in metabolism of LDL cholesterol in a gender-specific manner. The reason for the significant increase of serum LDL cholesterol level in DJ-1-knockout male mice may be as follows. First, the reduced level of DJ-1 inhibits transcription of the *LDLR* gene and renders the low level of LDLR as shown in Figure 1, resulting in inhibition of uptake of LDL, thereby increasing the serum LDL cholesterol level (Figure 8A). Second, endoplasmic reticulum (ER) stress induces the expression of transcription factor XBP1, which stimulates the expression of enzymes for fatty acid synthesis, including diacetylglicerol transferase-2 (Dagt2), stearyl CoA desaturase (scd1), acetyl CoA carboxylase (Acc2) and fatty acid synthase (Fasn). When these enzymes are lacking, serum LDL cholesterol level decreases [48]. Since DJ-1 represses ER stress [49], reduced or no expression of DJ-1 stimulates the expression of XBP1, thereby increasing serum LDL cholesterol level (Figure 8B). Third, it has been reported that several proteins harboring anti-oxidative activity lower the LDL cholesterol level [50]–[53]. Since DJ-1 has anti-oxidative stress function, reduced or no expression of DJ-1 may increase serum LDL cholesterol level (Figure 8C).

The effect of estrogen might explain why the serum LDL cholesterol level is not changed in DJ-1-knoout female mice. Estrogen increases the clearance of LDL cholesterol and then decreases serum LDL cholesterol level [54]–[56]. Estrogen directly stimulates promoter activity of the *LDLR* gene [40]. It is therefore thought that estrogen compensates the increased level of serum LDL cholesterol that was induced by DJ-1 knockout in female mice. Indeed, when DJ-1-knockout male mice were administered estrogen, the increased serum LDL cholesterol level in male mice was cancelled (Figure 7C). It has been reported that when LDLR-knockout mice were fed with a high-cholesterol diet, they had a three-fold higher concentration of the serum LDL cholesterol than that in mice fed with an ordinary diet, resulting in atherosclerosis [43]. Since the expression level of LDLR in DJ-1-knockout mice is lower than that in wild-type mice (Figure 1C), it is simply thought that the serum LDL cholesterol level of DJ-1-knockout mice is increased when DJ-1-knockout mice are fed with the high-cholesterol diet. While the serum LDL cholesterol level in wild-type mice was increased, there was no change of the serum LDL cholesterol level in DJ-1-knockout mice that had been fed with the high-cholesterol diet (Figure 7D). These results suggest that although DJ-1 significantly affects cholesterol homeostasis, there are many factors contributing to DJ-1′s effect on cholesterol homeostasis.

The *DJ-1* gene is the causative gene for familial Parkinson’s disease *park7*. It has been reported that the lower serum LDL cholesterol levels are associated with the onset of Parkinson’s disease [57]–[59]. The results in this study seem to be contradictory to those obtained by the cohort study of human cases as described above. Since DJ-1-knockout mice themselves do not show phenotypes of Parkinson’s disease [60], some compensation mechanisms might occur, thereby decreasing the effect of serum LDL cholesterol levels on the onset of Parkinson’s disease. It has been reported that simvastatin is associated with reduced incidence of dementia and Parkinson’s disease [61]. Simvastatin is a statin-related drug, and statins (3-hydroxy-3-methylglutaryl-coenzyme A reductase inhibitors) are a class of medications that reduce cholesterol by inhibiting 3-hydroxy-3-methylglutaryl-coenzyme A reductase. It would therefore be interesting to further analyze the effect of DJ-1 on metabolism of fatty acid.

## Materials and Methods

### Cells and Mice

NIH3T3 cells were purchased from American Tissue culture collection (ATCC). DJ-1-knockdown NIH3T3 (D2) cells described previously [62] and parental NIH3T3 cells were cultured in Dulbecco's modified Eagle's medium (DMEM) with 10% calf serum. DJ-1-knockout mice and normal mice were housed as described previously [60]. Originally established DJ-1-knockout mice were back-crossed more than 15 tomes and their genotype is now C57BL/6 background. C57BL/6 mice were used as control mice with *DJ-1* (+/+), and all of the mice were basically fed with normal diet (D12337, Research Diets, Inc. New Brunswick, NJ). Liver cell lines from DJ-1-knockout and normal mice were established as follows. Livers from newborn mice were cut out, digested with trypsin, and seeded on a 10-cm dish in DMEM with 10% calf serum. Cells were then transfected with an expression vector for T antigen of simian virus 40 (SV40), pMTI [63]. About two weeks after transfection, immortalized cells appeared and were cloned. All animal experiments were carried out in accordance with the National Institutes of Health Guide for the Care and Use of Laboratory Animals, and the protocols were approved by the Committee for Animal Research at Hokkaido University (the permit number 08–0468).

### RT-PCR

Nucleotide sequences of primers used for RT-PCR were as follows: mGAPDH 655-637: 5′-TGACCTTGCCCACAGCCTT-3′, mGAPDH 200-219: 5′-TCAACGGGAACGGGATCACC-3′, F-mLDLR: 5′-TGTGAATTTGGTGGCTGAAAAC-3′, R-mLDLR: 5′-AATAGGGAAGAAGATGGACAGGAAC-3′, mDJ-1 F: 5′-GCTTCCAAAAGAGCTCTGGTCA-3′, and mDJ-1 R: 5′-GCTCTAGTCTTTGAGAACAAGC-3′. Total RNAs were prepared from cells or mouse tissues and subjected to semi-quantitative RT-PCR analyses. PCR conditions were as follows: 1 min at 94°C, 30 sec at 94°C, 30 sec at 60°C and 22 cycles of 1 min at 72°C for GAPDH; 1 min at 94°C, 30 sec at 94°C, 30 sec at 60°C and 29 cycles of 1 min at 72°C for LDLR; and 1 min at 94°C, 30 sec at 94°C, 30 sec at 58°C and 26 cycles of 1 min at 72°C for DJ-1. After reactions, PCR products were extracted, separated on 1.4% agarose gels, and stained with ethidium bromide.

### Real-time PCR

Nucleotide sequences of primers used for real-time PCR were as follows: mLDLR-F: 5′-GAACTCAGGGCCTCTGTCTG-3′, mLDLR-R: 5′-AGCAGGCTGGATGTCTCTGT-3′, ACTB 412F: 5′-CCCTAAGGCCAACCGTGAAA-3′, ACTB 520R: 5′-ACGACCAGAGGCATACAGGGA-3′. Quantitative RT-PCR (real-time PCR) analyses were carried out as described previously [64].

### Luciferase Activity

Nucleotide sequences of oligonucleotides used for PCR primers to construct deletion mutants of promoter are as follows: Reverse LDLR: 5′-GGCCATGGTCACGACCTGCTGTG-3′, LDLR 225: 5′-GGAAGCTTAGCTCTTCACCGGCG-3′, LDLR 418: 5′-GGAAGCTTGTGGCGGAAGTTCCC-3′, LDLR897: 5′-GGAAGCTTCAGCCCTGTGTGGGG-3′, LDLR 1485: 5′-GGAAGCTTATCTGTCCAAGGCCG-3′, LDLR 1985: 5′-GGAAGCTTCGTTGCAGCAGCTCC-3′, LDLR 2944: 5′-GGAAGCTTCACTGCAAGCTCCGC-3′, and LDLR 3511: 5′-GGAAGCTTCTGCGCCACCACGCCT-3′. PCR products were digested with HindIII and NcoI and inserted into HindIII and NcoI sites of pGL4.10[luc2] (Promega, Madison, WI, USA). NIH3T3 and D2 cells in 24-well dishes were transfected with 0.75 µg of pGL4.10-hLDLR 200 or its deletion reporter plasmids and various amounts (0–1.0 µg) of pEF-DJ-1-HA together with 0.25 µg of pCMV-β-gal by the calcium phosphate method [65]. Two days after transfection, whole cell extract was prepared by addition of Triton X-100-containing solution from the Pica gene kit (Wako Pure Chemicals, Osaka, Japan) to the cells. About a one-fifth volume of the extract was used for the β-galactosidase assay to normalize the transfection efficiencies as described previously [7], and the luciferase activity due to the reporter plasmid was determined using a luminometer (Luminocounter Lumat LB 9507, EG & G Berthold, Bad Wildbad, Germany). Proteins in aliquots of the cell extract were analyzed by Western blotting with an anti-FLAG antibody (M2, Sigma, St. Louis, MO, USA) and visualized as described in the “Western blotting and antibody” section. The same experiments were repeated at least three times.

### Chromatin Immunoprecipitation (ChIP) Assay

ChIP assays using cultured NIH3T3 cells were performed according to the protocol of the ChIP Assay Kit (Millipore, Billerica, MA, USA). Briefly, after proteins had been cross-linked with DNA, cell pellets were resuspended in an SDS-lysis buffer and sonicated on ice using a sonicator (UR-20P, TOMY, Tokyo, Japan) 3 times for 20 sec each time. Genomic DNA was sheared to 300 to 1200 base pairs of length. Chromatin solution from 1 × 10^6^ cells/dish was preincubated with salmon sperm DNA and Protein A-agarose and incubated with species-matched IgG or with specific antibodies overnight at 4°C. DNA fragments immunoprecipitated were then used as templates for PCR with Ex taq (TaKaRa Bio, Kyoto, Japan) and reacted for 1 min at 94°C, 0.5 min at 94°C, 0.5 min at 72°C and 24 cycles of 30 sec at 72°C. Nucleotide sequences of oligonucleotide used for real-time PCR primers were as follows: ChmLDLR1-F: 5′-TCTGTGGGAGGAATTTGAGG-3′, ChmLDLR1-R: 5′-GTACTAGGGGCGAGGTTTCC-3′, ChmLDLR2-F: 5′- GTGTGGTGCAGGCCTTTAAT-3′, and ChmLDLR2-R: 5′- CCATCGTTGCTGGCTAGTTT-3′.

### Western Blotting and Antibodies

To examine the expression levels of proteins in cells, proteins were extracted from cells or mouse livers with a buffer containing 150 mM NaCl, 1 mM EDTA, 20 mM Tris (pH 8.0) and 0.5% NP-40. Proteins were then separated on a 12.5% polyacrylamide gel and subjected to Western blotting with respective antibodies. Proteins on the membrane were reacted with an IRDye 800- (Rockland, Philadelphia, PA, USA) or Alexa Fluor 680-conjugated secondary antibody (Molecular Probes, Eugene, OR, USA) and visualized by using an infrared imaging system (Odyssey, LI-COR, Lincoln, NE, USA). Antibodies used were anti-HA (1∶2000, MBL, Nagoya Japan), anti-SREBP-1 (1∶1000, Thermo Scientific, Waltham, MA, USA), anti-SREBP-2 (1∶1000, Abcam, Cambridge, UK), anti-actin (1∶4000, Chemicon, Temecula, CA, USA), anti-DJ-1 (1∶4000) and anti-LDLR (1∶1000, Abcam) antibodies. The rabbit anti-DJ-1 antibody was established by us as described previously [1].

### Gel-mobility Shift Assay

Gel mobility shift assays were carried out as described previously [66]. Briefly, a reaction mixture containing 10 µg of NIH3T3 cell nuclear extract, 2 µg/ml poly(dG–dC), 100 µg/ml bovine serum albumin, 16 mM Hepes (pH 7.9), 50 mM KCl, 4 mM EDTA, 0.8 mM DTT, 0.06% NP-40, 6% Ficoll 400 and an IRDye800-conjugated probe was incubated for 30 min at 4°C. DNA–protein complexes formed in the mixture were separated in a 4.5% polyacrylamide gel containing 0.25× TBE and visualized by an infrared imaging system (Odyssey, LI-COR). For a supershift experiment, the nuclear extract was first incubated with the IRDye800-conjugated probe as described above and then incubated with 1 µg of anti-DJ-1, anti-SREBP-1 (Thermo Scientific) and anti-SREBP-2 (Abcam) antibodies or non-specific IgG for 30 min at 4°C. Nucleotide sequences of oligonucleotides used for probes were as follows: SRE-EMSAs: 5′- GGGAAAATCACCCCATTGC-3′, mSRE-EMSAas: 5′- GGGAGCAATGGGGTGATTT-3′, mSREm-EMSAs: 5′- GGGAAATCGATGGATATGC-3′, and mSREm-EMSAas: 5′- GGGAGCATATCCATCGATT-3′.

### Co-immunoprecipitation Assay

Proteins were extracted from cultured cells by the procedure described previously [13]. Proteins were immunoprecipitated with a rabbit anti-DJ-1 antibody (1∶500) or normal IgG and precipitates were analyzed by Western blotting with anti-SREBP-1 (1∶1000, Thermo Scientific), anti-SREBP-2 (1∶1000, Abcam) or mouse anti-DJ-1 antibody (1∶1000, 3E8, MBL). Proteins on membranes were visualized as described above.

### Indirect Immunofluorescence

Mouse liver cells were fixed with 4% paraformaldehyde for 15 min and then with 0.1% Triton X-100 for 10 min, and reacted with an anti-LDLR antibody (1∶100, abcam) or with an anti-DJ-1 antibody (1∶500) for 2 hrs. The cells were then reacted with an FITC-conjugated anti-rabbit IgG or with a rhodamine-conjugated anti-rabbit IgG for 1 hr, and their nuclei were stained with DAPI. The cells were then observed under a fluorescent microscope (Biorevo BZ-9000, Keyence, Osaka, Japan).

### Tissue Preparation and Immunohistochemistry

Mice were perfused through the aorta with 1x PBS and then with a cold fixative consisting of 4% paraformaldehyde in PBS. After perfusion, the liver was quickly removed and post-fixed for overnight with 4% paraformaldehyde in PBS and then transferred to 10%, 20% and then 30% sucrose in PBS at 4°C for overnight. The liver pieces were cut into 10-µm-thick slices using a cryostat. Liver slices were treated with 0.1% Triton X-100 in PBS for 30 min and reacted with an anti-LDLR antibody (1∶100, abcam) or with an anti-DJ-1 antibody (1∶100) for 4 days at room temperature. After several washes, sections were reacted with an FITC-conjugated anti-rabbit IgG for 2 hrs at room temperature. The sections were also reacted with DAPI. Stained images were then observed under a fluorescent microscope (Biorevo BZ-9000).

### Measurement of Amounts of Total Cholesterol and LDL Cholesterol in Serum from Mice

After DJ-1-kockout mice and normal mice had been fasted for 14 hrs, they were killed and their serum was obtained. Amounts of total cholesterol and LDL cholesterol in serum were measured using cholesterol E and LDL-C.M. kits (Wako Pure Chemicals, Kyoto, Japan), respectively, according to manufacturer’s protocol. For administration of estrogen to DJ-1-kockout mice and normal mice, mice were subcutaneously injected with 5 µg/g body weight of estradiol dissolved in propylene glycol for every 6 days. After mice had been fasted for 15 hrs, LDL cholesterol in serum was measured. To examine the effect of high-fat diets, wild-type and DJ-1-kockout male mice at 13 weeks of age were fed with a high-cholesterol diet (D12336, Research Diets, Inc.), and their serum LDL cholesterol levels were measured as described above.

### Statistical Analyses

Data are expressed as means ± S.D or ± S.E for mouse experiments. Statistical analyses were performed using analysis of variance (one-way ANOVA) followed by unpaired Student’s *t*-test. For comparison of multiple samples, the Tukey-Kramer test was used.

## Supporting Information

Figure S1 **Association of DJ-1 and SREBP with the LDLR promoter.** Chromatin immunoprecipitation assays were carried out using chromatin prepared from NIH3T3 (A) and D2 (B) cells as described in Figure 5. Aliquots of immunoprecipitated DNA were separated on 1.4% agarose gels and stained by ethidium bromide.
(PDF)

Figure S2 **No direct binding of DJ-1 to SREBP-1 and SREBP-2.** ^35^S-labeled SREBP-1 and SREBP-2 were synthesized in vitro using the reticulocyte lysate of the TNT transcription-translation coupled system (Promega, Madison, WI). Labeled proteins were mixed with GST or GST-DJ-1 expressed in and prepared from *Escherichia coli* at 4°C for 60 min in a buffer containing 150 mM NaCl, 5 mM EDTA, 50 mM Tris (pH 7.5), 0.05% bovine serum albumin, and 0.1% Nonidet P-40 (NP-40). After washing with the same buffer, the bound proteins were separated in a 10% polyacrylamide gel containing SDS and visualized by fluorography.
(PDF)

Figure S3 **No direct binding of DJ-1 to the SRE.** Gel-mobility shift assays were carried out using nuclear extracts from SH-SY5Y cells and various amounts of purified human DJ-1 with IRDye800-labeled SRE as a probe.
(PDF)
