# Supplementary figures and images for: Transcriptional Activation of Low-Density Lipoprotein Receptor Gene by DJ-1 and Effect of DJ-1 on Cholesterol Homeostasis
Source: PLoS One. 2012 May 30;7(5):e38144. doi: 10.1371/journal.pone.0038144 (PMC3364227; doi:10.1371/journal.pone.0038144)

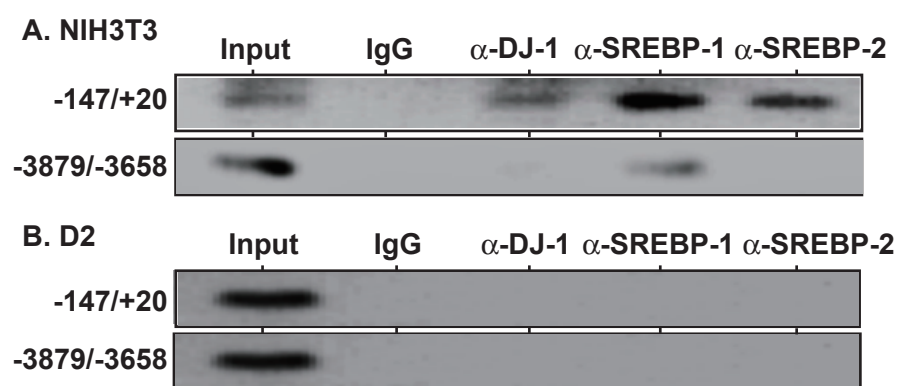

**Fig. S1**

Supplement: Figure S1 — Association of DJ-1 and SREBP with the LDLR promoter. Chromatin immunoprecipitation assays were carried out using chromatin prepared from NIH3T3 (A) and D2 (B) cells as described in Figure 5. Aliquots of immunoprecipitated DNA were separated on 1.4% agarose gels and stained by ethidium bromide. (PDF) [file pone.0038144.s001.pdf]

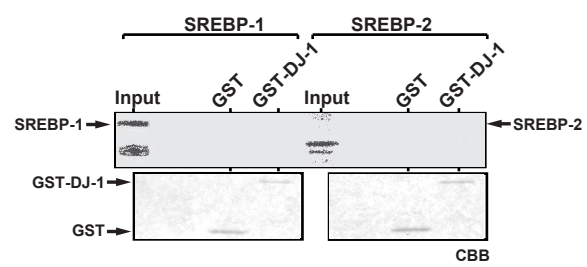

**Fig. S2**

Supplement: Figure S2 — No direct binding of DJ-1 to SREBP-1 and SREBP-2. 35S-labeled SREBP-1 and SREBP-2 were synthesized in vitro using the reticulocyte lysate of the TNT transcription-translation coupled system (Promega, Madison, WI). Labeled proteins were mixed with GST or GST-DJ-1 expressed in and prepared from Escherichia coli at 4°C for 60 min in a buffer containing 150 mM NaCl, 5 mM EDTA, 50 mM Tris (pH 7.5), 0.05% bovine serum albumin, and 0.1% Nonidet P-40 (NP-40). After washing with the same buffer, the bound proteins were separated in a 10% polyacrylamide gel containing SDS and visualized by fluorography. (PDF) [file pone.0038144.s002.pdf]

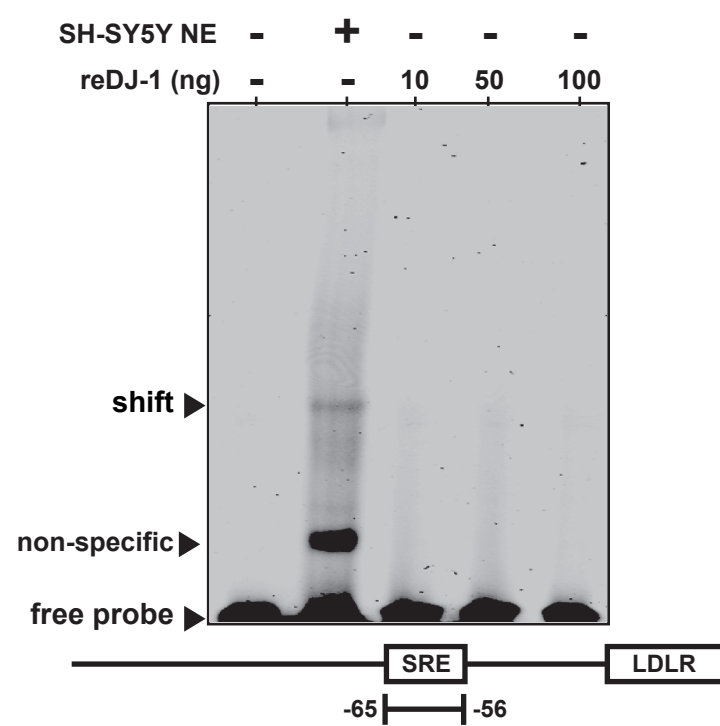

**Fig. S3**

Supplement: Figure S3 — No direct binding of DJ-1 to the SRE. Gel-mobility shift assays were carried out using nuclear extracts from SH-SY5Y cells and various amounts of purified human DJ-1 with IRDye800-labeled SRE as a probe. (PDF) [file pone.0038144.s003.pdf]
